# Remember the Drive Home? An Assessment of Emergency Providers' Sleep Deficit

**DOI:** 10.1155/2018/4501679

**Published:** 2018-01-23

**Authors:** Brian A. Ferguson, Hugh W. Shoff, Jennifer E. McGowan, Martin R. Huecker

**Affiliations:** Department of Emergency Medicine, University of Louisville, 550 S Jackson, Louisville, KY 40202, USA

## Abstract

**Objective:**

Sleep deprivation decreases work performance and predisposes workers to deleterious health outcomes. We sought to evaluate sleep hygiene and fatigue among emergency physicians.

**Methods:**

In March–June 2016, physicians and residents at an academic emergency medicine program were invited to complete a survey evaluating sleep and alertness.

**Results:**

Six attending physicians and 26 residents completed the survey. Among six personal priorities, sleep ranked fourth behind family, work, and leisure. 75% stated poor sleep impedes effectiveness as a physician while 53% noted difficulty falling asleep before a night shift. In the last three months, 39% of subjects forgot driving home from a shift, and 34% had fallen asleep while driving. 34% used medications to assist with sleep (including melatonin (36%), alcohol (27%), and prescription drugs (9%)). Most providers attested to phone (88%) and television exposure (69%) immediately prior to goal sleep onset.

**Conclusion:**

Despite sleep being identified as a priority among EM physicians, deleterious habits remain. Poor sleep affects perceived effectiveness and personal safety, as evidenced by a significant portion of providers falling asleep on the commute home. Night shift is the chief obstacle to optimal sleep hygiene.

## 1. Introduction

Sleep deprivation has long been recognized as a problem inherent to shift work [1–3]. Sleep deprivation predisposes workers to a range of deleterious health outcomes. Depression among medical staff is strongly associated with sleep deprivation [4]. Heart rate variability—a marker of sympathetic nervous system dominance and cardiovascular health—suffers after a night shift [5]. Sleep deficiencies related to shift work disrupt circadian cycles and lead to metabolic dysregulation, obesity, hypertension, and type II diabetes [2, 6, 7]. Additionally, shift working emergency physicians lose circadian rhythms of telomerase, portending greater aging effects [8]. Night shift specifically has been linked to increased risk for stroke and cardiovascular events [3, 9].

Sleep deprivation and its relationship with medical work performance have been a topic of interest across medical specialties. Concerns include decreased perceived patient safety, empathy, and motor coordination [10–14].

Despite the evidence for increased disease risk related to night shift and sleep deprivation, physicians seem to underappreciate the value of changing behavior [15]. Very little research has evaluated self-perceived sleepiness and shift alertness among emergency physicians and even less regarding coping strategies to mitigate limitations. The limited evidence suggests emergency physicians suffer from abnormal sleepiness and often inappropriate use of sleep aids [1, 16]. Our objective was to evaluate perceived sleepiness, physician health priorities, and use of wakefulness and sleep aids. In doing so, we sought to identify negative coping strategies and frequency of fatigue symptoms.

## 2. Materials and Methods

In an urban emergency medicine program, investigators surveyed residents and attendings to evaluate sleep barriers, rituals, and coping strategies. Variables of interest were adapted from prior survey-based evaluations of sleep in emergency providers [1, 16], as well as derived in house. The in-house variables included factors relating to alertness and perceived provider effectiveness—which became important for a future study performed months later, accessing real time alertness through a novel computerized shift assessment tool. The variables assessed time-off priorities, substance and medication use, perceived shift alertness, exposure to electronics, and other sleep hygiene components.

Prior evaluations of emergency providers utilized the Epworth Sleepiness Scale, including an assessment describing the ease at which providers fall asleep while stopping in traffic [1, 16]—which we adapted to falling asleep while driving and recollecting the commute home. Other borrowed variables assessed sleep latency [16], which was modified to more specifically identify shift type as associated with sleep latency. Additionally, respondents were surveyed on activities prior to the onset of goal sleep (e.g., alcohol use, sleep-aid medication, television, and light exposure), as well as pharmacologic and nonpharmacologic alertness aids—sleep aids comprising previous variables of interest [16].

This voluntary survey was completed using an online REDCap platform that ensured anonymous, unique, and independent survey results. Results were deidentified by the REDCap server, and data reductions were completed using REDCap database tools. Due to confidentiality constraints, resident and attending differences were not measured. The Institutional Review Board and the Human Subjects Protection Program Office approved the investigation.

Seventeen attending emergency physicians and 42 emergency residents were invited to participate. The assessment took place in March–June of 2016.

## 3. Results

Six attending physicians (response rate: 35%) and 26 residents (62%) completed the survey—total response rate: 32 of 59 (54%). Survey respondents were a mix of training levels: 44% first-year residents, 22% second year, 16% third year, and 19% attendings.

Among six personal priorities, sleep ranked 4th, behind family (1st), work (2nd), and leisure activities (3rd). Less important to providers were exercise and nutrition, in that order. Among the day (0800–1700), evening (1500–2400), and night shifts (2300–0800), subjects noted the greatest difficulty falling asleep before a night shift (53%), while 25% noticed difficulty falling asleep after a night shift. Subjects had the least difficulty falling asleep after a day shift (9%).

Among the three shifts, 91% of providers attested to feeling most fatigued during the night shift. In an independent assessment of four-hour time periods, the majority of providers attested to feeling most alert during the hours of 0800–1159 (31%) and 2000–2359 (28%) and least alert during 0400–0759 (75%). Overall, 75% felt sleepiness impedes effectiveness as a physician.

At some point in the last three months, 39% of subjects forgot the drive home from work; 3% stated they “fairly often” forgot the drive home. Thirty-four percent had fallen asleep while driving, over just a 3-month interval.

Regarding coping and mitigation strategies, 84% of subjects used medications (including caffeine) to increase alertness at work. Prescription alertness medications were used by 11%, while 34% used medications to assist with sleep (melatonin (36%), alcohol (27%), prescription drugs excluding melatonin (9%), and herbal supplements (9%)). Based on recollection, 75% of respondents reported an average sleep latency of 0–15 minutes.

In the last hour prior to sleep, 88% attested to phone exposure and 69% watched television. Sixty-six percent noted that their sleeping area has “some light” while only 34% attested to a sleep area that was “extremely dark.”

## 4. Discussion

Sleep was found to be a priority among EM physicians, and yet deleterious habits are pervasive. Poor sleep affects perceived provider effectiveness and personal safety, as evidenced by a significant portion (34%) of providers falling asleep on the commute home. These findings paralleled other evaluations of excessively sleepy emergency residents (38%) [16]. Night workers have up to a 30% incidence of experiencing excessive sleepiness or insomnia consistent with Shift Work Disorder, representing sleep wake schedule circadian mismatch [17].

Night shift is the chief obstacle to optimal sleep hygiene, and 91% of our providers attested to feeling fatigued during night shift. Other perceived sleepiness investigations have estimated an emergency medicine resident fatigue prevalence of 66% [1].

Night shift challenges have triggered relatively high levels of stimulant use while at work and, conversely, medications for sleep. Perhaps most concerning is that a large percentage of respondents regularly used alcohol for sleep onset. These findings reinforce previous evaluations estimating high rates of sleep onset alcohol use among emergency physicians [16]. Alcohol is a well-established barrier to quality sleep, resulting in delayed rapid eye movement sleep onset at all doses, and increasing sleep disruptions in the second half of a night's sleep [17].

Similar to prior studies evaluating night shift workers, emergency physicians experience circadian mismatch between endogenous rhythms and sleep wake/work cycles [18]. In our cohort, 53% noted greatest difficulty falling asleep before a night shift and least difficulty falling asleep after a day shift (9%). The observed circadian predisposition to feel most alert during the hours of 0800–1159, and least alert during 0400–0759, approximates diurnal activity and can make recovery time difficult. In an evaluation of working memory capacity linked with the sleep deficit of internal medicine residents, full recovery of working memory from sleep deficit did not occur until the fourth day of improved sleep [19]. As such, poor recovery on days off (manifesting as less than 6 hours of sleep) has been associated with poor quality of life among EM residents [1].

In the last hour prior to sleep, 88% attested to phone exposure, 69% watched television, and 66% reported “some light” in the sleeping area. All of the above behaviors interfere with natural melatonin secretion and predispose to circadian rhythm sleep disorders [20, 21].

Improved sleep can reverse harmful health consequences associated with sleep deprivation [9]. Rather than medicinal supplementation, strategic napping positively impacts emergency physician night shift performance—though this does not fully negate fatigue [22]. Unfortunately, sleep problems are pervasive among those employed in shift work regardless of the scheduling strategy applied [23]. The high prevalence of fatigue and deleterious habits observed in this study support that shift work fatigue and its mitigation will continue to be key areas of interest to the emergency physician.

### 4.1. Limitations

This is a study using data from a single program and thus represents a limited sample size. Residents in the study program work an average of 39.2 clinical hours per week, which is 14% less than the national average of 45.5 hours [24]. One might therefore predict relatively less physician fatigue by anticipating more time off and thus more time for restful sleep. Thus, while the rates of fatigue and physician impairment were quite high, they may underestimate the national average.

## 5. Conclusion

In this single center study, sleep was rated as a priority among emergency providers, and yet negative coping strategies are prevalent. Poor sleep affects perceived provider effectiveness and personal safety, as evidenced by a significant portion of providers falling asleep on the commute home. Night shift remains the chief obstacle to optimal sleep hygiene.
